# Clinico-mycological profile of tinea capitis and its comparative response to griseofulvin versus terbinafine 

**DOI:** 10.18502/cmm.5.1.532

**Published:** 2019-03

**Authors:** Ali Mikaeili, Hossein Kavoussi, Amir Hosein Hashemian, Mahdokht Shabandoost Gheshtemi, Reza Kavoussi

**Affiliations:** 1 Department of Mycology, School of Medicine, Kermanshah University of Medical Sciences, Kermanshah, Iran; 2 Department of Dermatology, Hajdaie Dermatology Clinic, Kermanshah University of Medical Sciences, Kermanshah, Iran; 3 School of Health, Kermanshah University of Medical Sciences, Kermanshah, Iran; 4 Student Research Committee, School of Medicine, Kermanshah University of Medical Sciences, Kermanshah, Iran

**Keywords:** Dermatophyte, Ectothrix, Endothrix, Griseofulvin, Terbinafine, Tinea capitis

## Abstract

**Background and Purpose:**

Tinea capitis is the most common superficial mycosis in children. This disease is a contagious infection with worldwide distribution and is occasionally associated with permanent alopecia. The treatment of this infection usually requires the administration of appropriate oral antifungal agents. The current study was conducted to evaluate the clinico-mycological profile of tinea capitis and compare the efficiency of oral griseofulvin and terbinafine in the treatment of this disease.

**Materials and Methods:**

This study was conducted on 69 patients, including 23 females (33.3%) and 46 males (66.7%), clinically suspected of tinea capitis. After the confirmation of tinea capitis diagnosis through direct examination, the subjects were randomly assigned into two groups of griseofulvin and terbinafine. Demographic data, clinical and mycological characteristics, and therapeutic outcome were recorded for both groups.

**Results:**

According to the results, tinea capitis was more common in children younger than 15 years (73.9%), athletes (37.7%), and males (66.7%), and those with frontal involvement (34.8%), non-inflammatory type (68.1%), endothrix (69.6%), and* Trichophyton tonsurans* species (41.7%). The griseofulvin and terbinafine groups had the treatment success rates of 90.9% and 80.6%, respectively (*P=0.311*). The griseofulvin group had a shorter therapeutic course than the terbinafine group (*P=0.129*).

**Conclusion:**

Although our findings demonstrated that both griseofulvin and terbinafine were effective in the treatment of tinea capitis, griseofulvin showed a little higher efficacy in this regard. Consideration of some variables, such as age, associated risk factors, clinical type, hair involvement pattern, and dominant pathogenic species, is important in the determination of the drugs.

## Introduction

Tinea capitis (TC) or scalp dermatophytosis is a highly contagious infection with worldwide distribution. This disease is the most common superficial mycosis in children [1, 2] and is still regarded as a major public health concern. The most important consequence of this infection is permanent hair loss, especially in untreated cases, as a result of a delay in treatment, inappropriate treatment, and inflammatory TC [3, 4]. In the recent years, new taxonomy was proposed for dermatophytes based on the internal transcribed spacer regions 1 and 2 of ribosomal DNA and partial large subunit, 60S ribosomal protein, fragments of β-tubulin, and translation elongation factor 3 [5].

Several studies in Iran have identified the dermatophyte species involved in dermatophytosis, including TC, based on the molecular methods using restriction fragment length polymorphism [6-8]. The treatment of TC requires the administration of oral antifungal drugs in order to penetrate the hair shaft. A variety of oral antifungal drugs have been used in the treatment of TC. Griseofulvin and terbinafine are the first and new antifungal drugs used for the treatment of superficial mycosis, respectively. These drugs have many advantages and limitations [9-15]. Given the changes in epidemiology, increased drug resistance, and lack of a similar study in this domain, the present study was conducted to determine the clinico-mycological characteristics of TC and appropriate drugs for the treatment of this infection based on clinical and mycological assessments.

## Materials and Methods

***Study design***

This cross-sectional randomized double-blind clinical trial was conducted on 80 patients with TC referring to Hajdaie Dermatology Clinic in the West of Iran over 2 years (i.e., 2015-2017). The patients were divided in two groups to receive oral griseofulvin (n=33) and terbinafine (n=36). The inclusion criterion was positive potassium hydroxide (KOH) examination in patients suspected of TC. On the other hand, the exclusion criteria were: 1) consumption of antifungal agents and systemic steroids in the recent 4 weeks, 2) immunodeficiency, 3) liver disease, 4) photosensitivity, 5) pregnancy or lactation, 6) failure to attend the follow-up visits, 7) consumption of other antifungal agents, and 8) emergence of side effects.

*Ethical consideration*

The present study was performed in accordance with the international ethical guidelines, consistent with the principles originating from the Helsinki Declaration on the biomedical research including human subjects. The study was approved by the Ethics Committee of the Kermanshah University of Medical Sciences, Kermanshah, Iran, and registered in the Iranian Registry of Clinical Trials database. Written informed consent was obtained from all participants or their parents before the initiation of the therapy. 

***Mycological examination***

The mycological examination was first performed using the hair and skin KOH examination. The patients with positive KOH results were enrolled in our study. The hair lesions were considered endothrix and ectothrix when observing arthroconidia inside and outside of the hair shaft, respectively. The samples were cultured and inoculated on Sabouraud medium agar slants with and without chloramphenicol cycloheximide adjunction and hold for 4 weeks at 25°C. Recognition of dermatophyte species was accomplished based on macroscopic and microscopic morphological characteristics. 

***Clinical examination***

The patients were divided into two groups of inflammatory (kerion) and non-inflammatory (gray patches and black dots) based on their clinical presentations. The lesions were scored based on such clinical findings as erythema, exudation, desquamation or scaling, and hair loss in moderate traction alopecia. Laboratory assessment, including complete blood count and liver function test, were performed at the baseline and 4 weeks after the treatment. 

According to the clinical findings, total sign score was determined before (i.e., at the baseline), during (i.e., in the second, fourth, and sixth weeks of treatment), and after (2 months post-treatment) the treatment. The total sign score included erythema or inflammation, scaling, and exudation or pustule. A significant reduction in the total sign score in weeks 2, 4, and 6, and month 2 was classified as early, timely, delayed, and very late response, respectively. In addition, poor response or no response was considered as treatment failure. On the other hand, an improvement in clinical signs and/or symptoms, along with negative KOH examination, was regarded as treatment. 

***Drug administration***

The guidelines of the European Society for Pediatric Dermatology were used in the TC treatment [15]. One group received oral griseofulvin at a dose of 20 mg/kg/day for 6-8 consecutive weeks. This group was advised to take the medication with high-fat foods for the desirable absorption of the antifungal drugs. instructed with high-fat foods. Oral terbinafine was administered at the doses of 62.5, 125, and 250 mg/day for the patients weighing < 20, 21-40, and > 40 kg, respectively, in the other group for 4-6 consecutive weeks. The patients with the inflammatory signs were prescribed oral antibiotics. Duration of treatment was based on the clinical response regarding the reduction of inflammation and improvement of other clinical findings.

***Statistical analysis***

The quantitative data were compared between groups using the independent t-test or Mann-Whitney U test. Furthermore, the analysis of the qualitative data was conducted by means of Chi-square and Fisher's exact tests. P-value less than 0.05 was considered statistically significant. Data analysis was performed in SPSS software (version 12).

## Results

Out of 80 patients, 69 cases, including 23 (33.3%) females and 47 (66.7%) males, completed the study. The mean age of the participants was 12.30±9.18 years, and the mean duration of infection was 5.28±2.38 weeks (Table 1). The lesions were located on the frontal, occipital, parietal, temporal, and vertex regions in 24 (34.8%), 13 (18.8%), 17 (24.6%), 11 (15.9%), and 4 (5.8%) cases, respectively (Table 1). The risk factors included playing sports, contact with an animal, a recent haircut at a suspicious barbershop, and use of swimming pool, which were reported in 26 (37.7%), 14 (20.3%), 10 (14.5%), and 1 (1.4%) cases, respectively. However, no risk factor was identified in 18 (26.1%) cases (Table 1).

Furthermore, inflammatory or kerion lesions, non-inflammatory lesions, gray patches, and black dots were observed in 22 (31.9%), 47 (68.1%), 36 (52.2%), and 11 (15.9%) patients, respectively (Table 1). The KOH examination revealed endothrix and ectothrix in 48 (69.6%) and 21 (30.4%) patients, respectively (Table 1). The results of culture revealed the presence of *Trichophyton tonsurans, T. verrucosum, *and* T. menagrophytes* in 15 (21.7%), 11 (15.9%), and 3 (4.3%) patients, respectively. In addition, *Microsporum gypseum* and *M. canis* were detected in 5 (7.2%) and 2 (2.9%) cases, respectively. However, the culture was negative in 33 (47.8%) cases (Table 1).

Out of 80 patients, 69 cases, including 33 patients (females: 10 [30.3%] and males: 23 [69.7%]) in the griseofulvin group and 36 patients (females: 13 [36.1%] and males: 23 [63.9%]) in the terbinafine group, completed the study and received 8 weeks of treatment and follow-up (Table 1). There was no statistically significant difference between the two group in terms of age (*P=0.871*), gender (*P=0.799*), duration of infection (*P=0.213*), location of lesion (*P=0.315*), risk factors (*P=0.475*), type of lesion (*P=0.794*), clinical type (*P=0.612*), KOH examination (*P=0.982*), culture results (*P=0.988*), treatment outcome (*P=0.311*), and therapeutic course (*P=0.129*; Table 1). Furthermore, 30 (90.9%) and 29 (80.6%) patients in the griseofulvin and terbinafine groups were successfully treated. However, the results indicated no statistically significant difference between the two groups in this regard (*P=0.311*; Table 1).

Based on the results, 14 (42.4%), 13 (39.4%), 3 (9.1%), and 0 (0.0%) cases in the griseofulvin group showed early, timely, delayed, and very late responses to treatment, respectively. Regarding the terbinafine group, 9 (19.4%), 14 (38.9%), 6 (16.7%), and 1 (2.8%) patients demonstrated early, timely, delayed, and very late clinical responses to treatment, respectively (*P=0.129*). There was no significant difference between treatment outcome and age (*P=1*, *P=0.686*), gender (*P=1*, *P=0.225*), duration of involvement (*P=0.601*, *P=0.674*) , KOH examination (P=0.329, *P=0.466*), type of lesion (*P=0.534*, *P=0.958*), clinical type (*P=0.212*, *P=0.927*), and culture results (*P=0.212*, *P=0.315*) in both groups (Table 2).

**Table 1:** Demographic and clinico-mycological profile of the patients and treatment outcome in the two groups

**Variables **	**G** **riseofulvin group**	**Terbinafine group**	***P-value***
Mean age (year)	11.63±6.8	12.94±10.32	0.871
Gender n (%)			
Female Male	10 (30.3%) 23 (69.7%)	13 (36.1%) 23 (63.9%)	0.799
Mean duration of involvement (week)	4.69±1.81	5.83±3.09	0.213
Location of lesions n (%)			
Frontal Occipital Parietal Temporal Vertex	11 (33.3%) 3 (9.1%) 11 (33.3%) 6 (18.2%) 2 (6.1%)	13 (36.1%) 10 (27.8%) 6 (16.7%) 5 (13.9%) 2 (5.6%)	0.315
Risk factor n (%)			
Sport Contact with animals Recent haircut Use of swimming pool Indeterminate	12 (36.4%) 7 (21.2%) 3 (9.1%) 0 (0%) 11 (33.3%)	14 (38.9%) 7 (19.4%) 7 (19.4%) 1 (2.8%) 7 (19.4%)	0.475
Type of lesion n (%)			
Inflammatory Non-inflammatory	12 (36.4%) 21 (63.6%)	10 (27.8%) 26 (72.2%)	0.794
Clinical type n (%)			
Gray patch Kerion Black dot	17 (51.5%) 12 (36.4%) 4 (12.1%)	19 (52.8%) 10 (27.8%) 7 (19.4%)	0.612
KOH examination n (%)			
Ectothrix Endothrix	10 (30.3%) 23 (69.7%)	11 (30.6%) 25 (69.4%)	0.982
Culture n (%)			
*T. tonsurans * *T. verrucosum* *T. menagrophytes* Nannizzia gypsea (formerly M. gypseum) Arthroderma otae (formerly *M. canis*) Negative	8 (24.2%) 5 (15.2%) 1 (3.0%) 2 (6.1%) 1 (3.0%) 16 (48.5%)	7 (19.4%) 6 (16.7%) 2 (5.6%) 3 (8.3%) 1 (2.8%) 17 (47.2%)	0.988
Treatment outcome n (%)			
Success Failure	30 (90.9%) 3 (9.1%)	29 (80.6%) 7 (19.4%)	0.311
Clinical response to treatment n (%)			
Early Timely Delayed Very late Failure	14 (42.4%) 13 (39.4%) 3 (9.1%) 0 (0.0%) 3 (9.1%)	9 (25.0%) 14 (38.9%) 5 (13.9%) 1 (2.8%) 7 (19.4%)	0.129

T: *Trichophyton*, M: *Microsporum*

**Table 2:** Treatment outcome of tinea capitis based on demographic, clinical, and mycological characteristics in the two groups

	**G** **riseofulvin group**		***P-value***	**Terbinafine group**		***P-value***
	**Cure**	**failure**		**Cure**	**failure**	
Age						
<8 years n (%) ≥8 years	15 (93.8%) 15 (88.2%)	1 (6.3%) 2 (11.8%)	1	10 (76.9%) 19 (82.6)	3 (23.1%) 4 (17.4%)	0.686
Gender no (%)						
Female Male	9 (90.0%) 21 (91.3%)	1 (10.0%) 2 (8.7%)	1	9 (69.2%) 20 (87.0%)	4 (30.8%) 3 (13.0%)	0.225
Duration of involvement n (%)						
<4 weeks ≥4 weeks	16 (94.1%) 14 (87.5%)	1 (5.9%) 2 (12.5%)	0.601	13 (86.7%) 16 (76.2%)	3 (3.3%) 5 (23.8%)	0.674
KOH examination n (%)						
Ectothrix Endothrix	10 (100.0%) 20 (87.0%)	0 (0.0%) 3 (13.0%)	0.329	10 (90.9%) 19 (76.0%)	1 (9.1%) 6 (24.0%)	0.466
Type of lesion n (%)						
Inflammatory Non-inflammatory	12 (100.0%) 18 (85.7%)	0 (0.0%) 3 (14.3%)	0.534	8 (80.0%) 21 (80.8%)	2 (20.0%) 5 (19.2%)	0.958
Clinical type n (%)						
Gray patch Kerion Black dot	14 (82.4%) 12 (100.0%) 4 (100.0%)	3 (17.6%) 0 (0.0%) 0 (0.0%)	0.212	15 (78.9%) 8 (80.0%) 6 (85.7%)	4 (21.1%) 2 (20.0%) 1 (14.3%)	0.927
Culture n (%)						
*Trichophyton* species *Microsporum* species	14 (100.0%) 2 (66.7%)	0 (0.0%) 1 (33.3%)		12 (80.0%) 4 (100.0%)	3 (20.0%) 0 (0.0%)	

## Discussion

This study demonstrated that oral griseofulvin resulted in a higher rate of TC treatment and more rapid clinical improvement than oral terbinafine; however, this difference was not statistically significant. Our results showed that TC was more common in children (mean age of 12.30 years), males (66.7%), and athletes (37.7%), as well as in those with frontal involvement (34.8%), non-inflammatory lesion (68.1%), gray patches (52.2%), endothrix hair involvement (69.6%), *T. tonsurans* species (41.7%), and mean infection duration of 5.28±2.38 weeks.

Consistent with our findings, in a couple of studies performed in Iran, TC was reported to be more common in the children aged up to 10 years (6, 7). It is assumed that children, especially boys, have a higher tendency to sport, contact with animals, low attention to personal hygiene, and such occupations as shepherding. In a study carried out in Egypt, the parietal region was the most common spot of involvement [16]. Nonetheless, in a study conducted in Brazil [17] on the micro-epidemic of TC, the frontal region was the most commonly affected location, which is consistent with our findings. The location of involvement can be related to the type of recreation, tendency to a specific sport, contact with animals, and occupation at any geographical sites, resulting in contact with dermatophytes. 

In the current study, the results of direct examination revealed that endothrix (69.6%) was more common than ectothrix (30.4%). While some studies [15, 18] have reported endothrix to be more common than ectothrix, others have demonstrated the higher prevalence of ectothrix [11, 19] based on KOH examination. This discrepancy could be related to the difference in the dominant dermatophyte species in various geographic areas. In this regard, direct examination has indicated that while *T. tonsurans* is the dominant species of endothrix [20] in one area, *M. canis* is the dominant species leading to ectothrix in another area [17]. In the current study,* T. tonsurans* (41.7%), followed by *T. verrucosum* (30.5%), were identified as the most frequent causative agents. 

In a number of studies performed in Iran, Didehdar [6], Abastabar [7], and Mohammadi [8] reported *T. tonsurans, T. interdigitale, *and* M. canis *as the most common frequent dermatophytes in TC, respectively. In a study carried out in a rural region in southern Ethiopia among schoolchildren, *T. verrucosum* was the most common pathogen of TC [21]. Identification of dominant pathogens of TC in each area is a matter of paramount importance in the selection of the proper treatment. 

The anthropophilic dermatophyte species induce mild inflammation, while the zoophilic dermatophyte species induce severe inflammation in TC. However, a number of anthropophic dermatophyte species can induce severe inflammation and kerion lesions [20, 22]. Inflammatory TC has been reported to be a rare condition in the majority of studies [16, 20, 23]. Nonetheless, this infection (31.9%) was common among our patients. This may be due to the fact that our patients had contacts with animals provoking changes in the pattern of anthropophilic dermatophytes species for the induction of severe inflammation [20, 22]. 

Our findings revealed sport (37.7%) and contact with an infected animal (20.3%) as the most common risk factors for TC. These factors, along with other factors, such as high-risk jobs, use of public swimming pool, and nonpractice of personal hygiene (e.g., sharing hairbrushes, hat, headrest, and bed) are effective not only in the induction of TC but also in the determination of the disease clinical type and location of lesions [22, 24, 25].

The most important factors in the prevention of dermatophytosis and permanent hair loss, particularly inflammatory TC, include the early referral to the physician, accurate diagnosis, and appropriate antifungal treatment [11, 22]. In our patients, the mean interval of referral to the physician was obtained as 5.28±2.38 weeks. Based on the results, people education, especially in high-risk areas, physician’s familiarity with TC, proper facilities for patient’s referral to a therapeutic center, and appropriate treatment are important factors for the prevention of TC and avoidance of the spread of scarring alopecia. 

The in vitro antifungal susceptibility of clinical dermatophytes is very important for the determination of the proper drug. Afshari et al. [26] showed that griseofulvin and terbinafine were highly effective against T. verrucosum. Furthermore, Falahati et al. *[*27*]* indicated T. interdigitale was highly susceptible to griseofulvin and terbinafine with a minimum inhibitory concentration (MIC) of 0.0312 μg/ml, followed by T. verrucosum (MIC=0.008 μg/ml). In a study presenting T. interdigitale as the most common isolate*, *terbinafine (MIC=0.002-0.25 μg/ml) was reported as the most potent antifungal drug against all isolates [28]. Based on the majority of the studies, terbinafine is a well-tolerated, efficacious, and first-line drug for the treatment of TC. This drug is especially preferred when *Trichophyton *genus accounts for the incidence of TC [9, 14, 29, 30]. 

Griseofulvin has been reported as an effective and cost-efficient drug in the treatment of TC by a number of studies [11, 23, 28]. Chen [9] and Tey [29] concluded that griseofulvin is a standard drug when TC is caused by *Microsporum* genus. However, Grover [11] and Bhanusali [31] concluded that griseofulvin is efficacious if TC results from *Trichophyton *genus among *T. violaceum *and* T. tonsurans.*

**In a meta-analysis targeted toward the investigation of several randomized controlled trials comparing griseofulvin and terbinafine in the treatment of TC, these drugs showed different efficacies when considering fungal species. However, in another study, no significant difference was reported in the overall efficacy of the two drugs at a specific dose [** 10 **]. Based on the direct examination,** ***T. tonsurans*** **species, gray patch, and endothrix feature were the most frequent findings in our patients, among whom oral griseofulvin had a higher treatment success rate than terbinafine** **(90.9% vs. 80.6%).**

With respect to the pharmacokinetic characteristics of terbinafine, its high concentration in sebum, and its undetectability in sweat and given the fact that the sebaceous glands do not develop until puberty, terbinafine is less effective in children with ectothrix TC [13]. According to our findings, most of the patients were children with endothrix, this explains why griseofulvin was more efficacious than terbinafine in our study.

In our patients, the early improvement of clinical signs was achieved in 42.2% and 25.0% of the patients in the griseofulvin and terbinafine groups, respectively. In a couple of studies, griseofulvin resulted in an earlier improvement of TC clinical signs than terbinafine [13, 32]. Griseofulvin has anti-inflammatory properties and results in the inhibition of proliferation, glycosaminoglycan secretion, and protein synthesis in fibroblasts [33]. Due to the antifungal and anti-inflammatory properties of griseofulvin, this drug could be the best agent for the treatment of TC, especially its inflammatory type. 

## Conclusion

Based on the results of direct examination, TC was most often observed in boys, and those with frontal location involvement, endotrix, gray patch, and *T. tonsurans* as the prominent pathogen. In addition, griseofulvin resulted in a high treatment success rate and earlier clinical response than terbinafine; however, this difference was not statistically significant. With respect to the high incidence of TC in athletes and patients with lower age, gray patch, endothrix, and *T. tonsurans* as the prominent pathogen, griseofulvin was the first-line drug for the treatment of TC.

Therefore, it is important to consider some aspects, such as patient’s age, clinical type, KOH examination results, risk factors, and prominent pathogen in the geographic site for the selection of the appropriate drug for treatment of TC. It is suggested to perform further multi-center studies using larger sample size and molecular assessments for the detection of dermatophyte and in vivo investigations to identify species sensitivity and proper antifungal drugs. 
